# Crystal structure of MOA in complex with a peptide fragment: A protease caught *in flagranti*

**DOI:** 10.1016/j.crstbi.2020.04.003

**Published:** 2020-04-22

**Authors:** Dipankar Manna, Gabriele Cordara, Ute Krengel

**Affiliations:** a Department of Chemistry, University of Oslo, PO Box 1033 Blindern, 0315, Oslo, Norway

## Abstract

The *Marasmius oreades* agglutinin (MOA) is the holotype of an emerging family of fungal chimerolectins and an active Ca^2+^/Mn^2+^-dependent protease, which exhibits a unique papain-like fold with special active site features. Here we investigated the functional significance of the structural elements differentiating MOA from other papain-like cysteine proteases. X-ray crystal structures of MOA co-crystallized with two synthetic substrates reveal cleaved peptides bound to the catalytic site, corresponding to the final products of the proteolytic reaction. Anomalous diffraction data on crystals grown in the presence of calcium and manganese, cadmium or zinc resolve the calcium/manganese preference of MOA and elucidate the inhibitory roles of zinc and cadmium towards papain-like cysteine proteases in general. The reported structures, together with activity data of MOA active site variants, point to a conservation of the general proteolysis mechanism established for papain. Ultimately, the findings suggest that papain and the papain-like domain of MOA are the product of convergent evolution.

## Highlights

• MOA is a chimerolectin with protease activity carrying a unique papain-like fold.
• The structures of inactive MOA variants reveal cleaved peptides in the active site.
• Anomalous diffraction data elucidate activation or inhibition by different metals.
• The MOA papain-like domain is a product of convergent evolution.
• MOA is the holotype of an emerging family of enzymatically active fungal lectins.

## Graphical abstract

**Figure:**
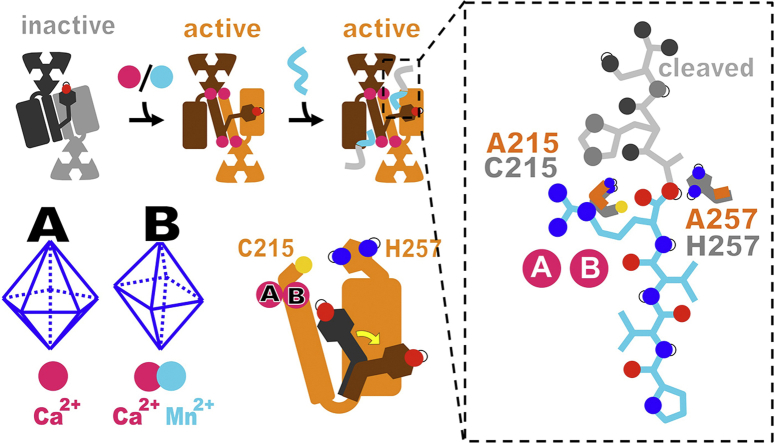

## 1 Introduction

**Fig. 1:**
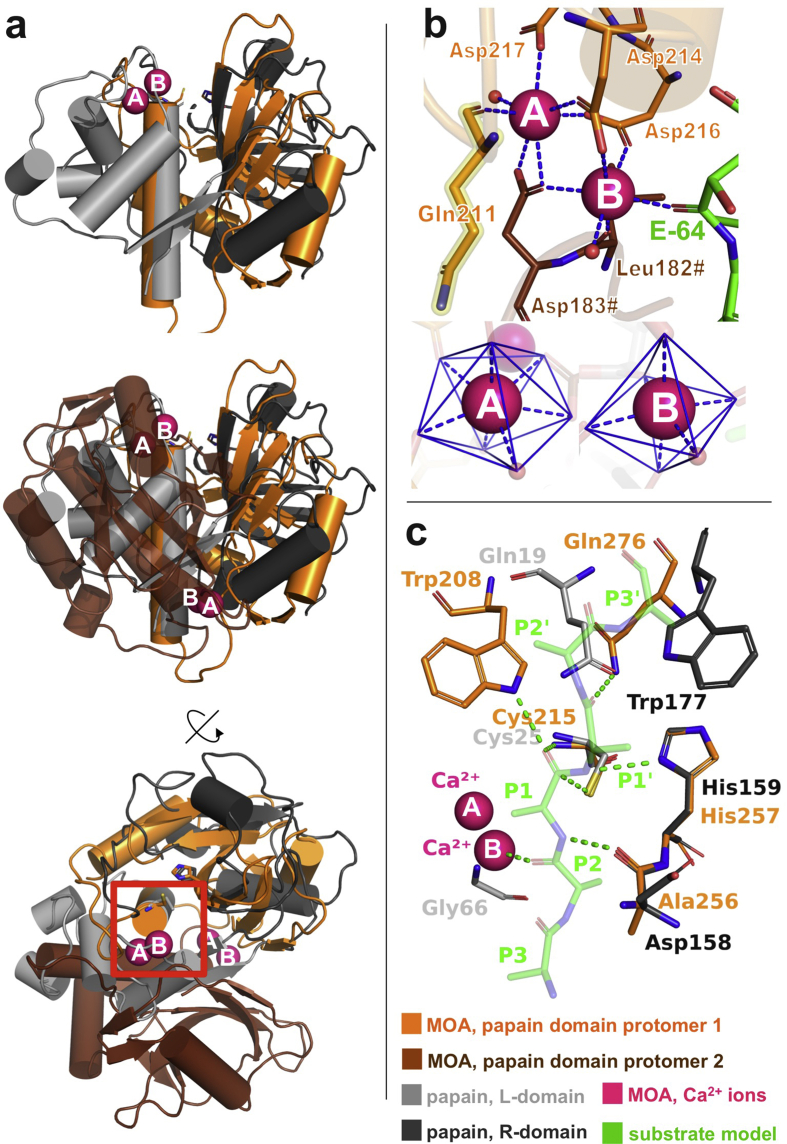
Unique features of MOA papain-like domain. (a) Superimposition of MOA (orange/brown, PDB ID: 3EF2 (Grahn et al., 2007)) and papain (grey, PDB ID: 1CVZ (Tsuge et al., 1999)), with the L- and R-domains shown in light and dark grey, respectively. In MOA, the α-helical elements of the L-domain of papain are replaced by a dimerization interface. The upper panel only depicts one protomer of MOA (orange), the lower panels include both subunits (orange/brown) and two active sites (one of them marked with a red square and detailed in panel b). (b) Binuclear metal-binding cluster of MOA (PDB ID: 5MU9 (Cordara et al., 2017)), with sites A and B displaying pentagonal bipyramidal and octahedral coordination, respectively. Note metal-A coordinating residue Gln211 (highlighted in yellow), which exhibits less favorable torsion angles, at the border of allowed regions in the Ramachandran plot. (c) Superimposition of the active sites of MOA (orange) and papain (light/dark grey), with a peptide substrate (green) modeled into the MOA binding site according to (Cordara et al., 2016). Papain residues Gln19 and Trp177 are replaced by Trp208 and Gln276 in MOA, representing a structural swap.

Papain-like cysteine proteases (PLCPs, EC 3.4.22) form the cysteine protease superfamily with the largest number of members (Turk et al., 1997, Brömme, 2001, Lecaille et al., 2002). PLCPs are found in bacteria, viruses, plants and animals, and are involved in a number of physiological and pathological processes (Dubey et al., 2007). PLCPs play a role in antigen presentation (Dubey et al., 2007), cancer, inherited diseases, parasitic infections (Rzychon and Chmiel, 2004) and host defense (O'Farrell and Joshua-Tor, 2007, Smith et al., 1976). Papain-like proteases are characterized by a conserved α+β folding motif, commonly referred to as the “papain fold”. The papain fold comprises two subdomains, known as L(left)- and R(right)-domains (Drenth et al., 1968, Heinemann et al., 1982). The interface between the two subdomains forms a V-shaped groove, at the bottom of which lies the catalytic center (Drenth et al., 1968, Turk and Gunčar, 2003) (Fig. 1a; see Fig. S1 for superimposition of structural motifs).

The *Marasmius oreades* agglutinin (MOA) is the best-studied representative of a novel family of fungal chimerolectins that exhibits a unique variation of the papain fold (Winter et al., 2002, Tateno and Goldstein, 2004, Grahn et al., 2007). While acting as a dimerization domain, the papain-like domain of MOA retains the characteristic L-/R-domain partition of PLCPs. In a remarkable example of resource optimization, the L-domain is composed of structural elements borrowed from the L-domain of the other protomer, reconstituting the full functional unit. As additional feature, the MOA dimer carries two binuclear metal-binding clusters, symmetrically placed at the dimerization interface (Grahn et al., 2009). Each cluster offers two different coordination geometries, pentagonal bipyramidal (site A) and octahedral (site B) (Grahn et al., 2009) (Fig. 1b). MOA requires the presence of calcium or manganese (II) to switch from the inactive form to an active protease (Cordara et al., 2011, Wohlschlager et al., 2011). Metal ion binding triggers a conformational rearrangement at the active site of MOA, opening it for catalysis (Cordara et al., 2017). While other divalent cations cannot functionally replace calcium or manganese, cadmium or zinc inhibit the enzymatic activity of MOA (Cordara et al., 2011). The latter is a known effect of zinc on papain and related enzymes (Sluyterman and Wijdenes, 1976, Maret, 2013). The catalytic cleft of MOA has been mapped through the use of suicide inhibitors (Cordara et al., 2016, Cordara et al., 2017). Two residues involved in the formation of the oxyanion hole and in substrate coordination (Trp208 and Gln276, respectively) are found in swapped positions and roles compared to other papain-like enzymes (Fig. 1c) (Storer and Ménard, 1994). Additionally, the substrate is in direct contact with the octahedrally coordinated metal ion (site B, Fig. 1c), hinting at its possible involvement in catalysis.

Here we present the X-ray crystal structure of enzymatically inactive MOA variants co-crystallized with two synthetic peptides mimicking proteolytic substrates. Furthermore, we report functional and structural data on MOA variants targeting Trp208 and Gln276. Finally, we collected anomalous diffraction data on MOA crystals grown in the presence of calcium, manganese (II), zinc and cadmium. Overall, the data confirm the substrate binding geometry postulated in previous publications and the strong preference of site A for calcium (Cordara et al., 2016, Cordara et al., 2017), whereas site B is found to be more promiscuous. Furthermore, zinc and cadmium inhibit the activity of MOA by forming a tight complex with the catalytic cysteine (Cys215) and histidine (His257), as predicted for papain (Maret, 2013). The peptide complex structures show well-defined electron density accounting for the N-terminal fragment of the substrate mimics after proteolytic cleavage, representing one of few examples of PLCP structures in complex with a proteolysis product. The structural and functional data collected on MOA are consistent with the well-studied two-step reaction mechanism of papain-like proteases, and additionally reveal new insight into the evolutionary origins of this intriguing family of fungal chimerolectins.

## 2 Materials and methods

### 2.1 Expression and purification

Starting from an IPTG-inducible pT7 vector containing the cDNA for wild-type MOA (MOApT7-LO, described by Cordara et al. (2011), a mutation was introduced at different codons by site-directed mutagenesis using the QuikChange II kit (Stratagene). Following the protocol provided by the manufacturer, single-site functional variants were generated for codons 215 (C215A), 208 (W208A, W208Q), 257 (H257A) or 276 (Q276A, Q276W). Double-site variants were generated using the same protocol and starting from one of the previous constructs (C215A/H257A from C215A; W208Q/Q276W from W208Q). Expression of the metal-free form of all functional mutants and the wild-type enzyme was carried out in *Escherichia coli* BL21 (DE3) cells transformed with the corresponding expression vector and following the protocol described by Cordara et al. (2011). Bacterial cell pellets were collected by centrifugation and stored at −80 °C before lysis. After thawing, the bacterial pellets were resuspended in a lysis buffer containing 50 mM Tris/HCl pH 8.0, 0.15 M NaCl, 2 mM EDTA, 1× concentrated c*O*mplete protease inhibitor cocktail EDTA free (Roche Diagnostics Ltd), 1 μl/ml Benzonase nuclease (Thermo Scientific) and 4 mg/ml hen egg white lysozyme. After incubation on a shaker for 2 h at RT, the insoluble fraction was removed by two rounds of centrifugation (20,000 rcf, 45 min). The clarified cell lysate was passed through a d-Gal-sepharose affinity column (Thermo Scientific), followed by extensive washing with 20 mM imidazole/HCl pH 8.0 buffer and elution of the protein using a 1.0 M d-Gal single step gradient. The fractions containing MOA were concentrated using a 10,000 MWCO PES membrane (Vivaspin, Sartorius AG) to a volume of ~500 μl. Polishing of the protein preparation was carried out by size exclusion chromatography (SEC) using a Superdex 75 10/300 GL gel-filtration column (Tricorn, GE Healthcare Life Sciences) and a buffer containing 20 mM imidazole/HCl pH 8.0, 2 mM EDTA, 0.2 M d-Gal, 0.15 M NaCl and 2 mM DTT. The fractions containing the purified protein were pooled, concentrated to a final protein concentration of 10–15 mg/ml using concentrator tubes with a 10,000 MWCO PES membrane (Vivaspin, Sartorius AG) and underwent three rounds of buffer exchange against a 20 mM imidazole/HCl pH 8.0, 2 mM EDTA, 2 mM DTT. Cell lysis and protein purification were carried out following the same protocol described for MOA by Cordara et al., 2011, Cordara et al., 2014.

### 2.2 Enzyme activity assay

MOA activity was tested using native α1-antitrypsin (α1-AT) from human plasma (Sigma–Aldrich) as the target substrate, as described in (Cordara et al., 2011). A reaction mixture containing 0.2 mg/ml α1-AT, 50 mM Na-HEPES pH 7.5, 10 mM DTT, 10 mM CaCl_2_ and 0.2 mg/ml MOA was incubated at 37 °C for 24 h. The reaction was stopped by adding 5 μl of SDS-PAGE sample buffer 4× to 15 μl of the reaction mixture and boiling at 100 °C for 10 min. Samples were analyzed by SDS-PAGE on a 4–12% NuPAGE gel and stained with Coomassie brilliant blue dye. Gel images were analyzed using the Fiji distribution of the ImageJ manipulation software (Schindelin et al., 2012).

### 2.3 Thermal stability assay

The method described by Ericsson et al. (2006) and Niesen et al. (2007) was adapted into a custom protocol, which uses SYPRO Orange (Sigma) as the fluorescent probe (Hawe et al., 2008). The thermal stability of wild-type MOA and its functional variants was tested in a reaction mixture containing a 0.1 M Na-HEPES pH 7.5, 0.15 M NaCl, the SYPRO Orange dye diluted 1:1000 from the commercially available stock solution, and the protein at a final concentration of 0.1 mg/ml. Fluorescence-compatible 96-well RT-PCR plates (Roche) were filled with a final volume of 25 μl reaction mixture/well. The fluorimetric run was performed increasing the temperature from 20 °C to 95 °C at a speed of 1 °C/min on a LightCycler 480 thermocycler (Roche), probing the samples with an excitation wavelength of 483 nm and reading the fluorescence at 568 nm. The fluorescence data were processed using a custom version of the Microsoft Excel-based DSF Analysis tool provided by Frank Niesen (Structural Genomics Consortium, Oxford, United Kingdom). The melting temperature was calculated from the DFS Analysis output through the software GraphPad Prism, version 5 (GraphPad Software Inc., La Jolla, CA, United States of America).

### 2.4 Crystallization

MOA crystals grew from a solution containing the purified protein at a concentration of 5 mg/ml and the trisaccharide Galα1,3(Fucα1,2)Gal (Dextra; 1:20 MOA:sugar molar ratio), pre-mixed directly before the experiments were set up. Hanging-drop vapor diffusion experiments were carried out using 24-well tissue culture plates (TPP, Sigma–Aldrich) and siliconized glass cover slides (Hampton Research), on which 1 μl crystallization solution was added to an equal volume of the protein-ligand solution. Crystals of MOA in complex with peptide substrates PVPRAHS or PVVRAHS (GenScript, USA) were grown according to the same protocol detailed above, pre-mixing sugar and protein, and additionally adding one of the peptides at a 1:10 MOA:peptide molar ratio. Details of the crystallization conditions are reported in Table S1. Rod-shaped crystals grew over the course of 2–4 weeks at 20 °C, with dimensions of 0.05 mm × 0.05 mm × 0.4 mm. Crystals were subsequently cryoprotected in mother liquor supplemented with 15% ethylene glycol and flash-cooled in liquid nitrogen for data collection.

### 2.5 Data collection, processing, scaling and structure determination

Diffraction data were collected at the European Synchrotron Radiation Facility (Grenoble, France) at beamlines ID29 (Pilatus 6M detector), ID23-1 (ADSC Quantum Q315r detector) and ID23-2 (MAR225 detector). To mitigate the effect of radiation damage, we employed a helical data collection strategy. Anomalous diffraction data for crystals grown in the presence of manganese chloride were collected at beamline ID23-1 around the manganese Κ absorption edge. Diffraction data for crystals grown in the presence of zinc or cadmium were collected at beamline ID29 at 9.7 keV, which corresponds to a high-energy remote point to the K-edge of zinc (9.66 keV), and at the lowest energy available at the beamline (6.0 keV), respectively.

X-ray data were processed and scaled using the XDS software package (Kabsch, 2010). Anomalous diffraction data were collected on different parts of the same crystal for each of the four data sets (Mn, Ca/Mn, Zn, Cd). They were moreover integrated over the same resolution range using XDS and placed on a common scale with XSCALE (Kabsch, 2010). Scaling statistics are given in Table 1, Table 2 (anomalous data). Crystals belonged to space group *P*6_3_22, with cell parameters of approximately *a* = 121.0 Å, *b* = 121.0 Å, *c* = 100.0 Å. The structures were solved by molecular replacement with the software PHASER (McCoy et al., 2007), using as search models a modified the calcium-bound structure of MOA (PDB ID: 3EF2 (Grahn et al., 2009)) lacking the Pro54-Val56 loop and with residues showing flexible or generally poorly defined side chains mutated to Ala. PHASER found a solution for a single MOA protomer in the asymmetric unit, with m*F*o-D*F*c maps showing well-defined, positive density at the binuclear metal binding site, two or three of the sugar binding sites and in the active site cleft.

**Table 1:** Data collection and refinement statistics.^g^

	PVPRAHS	PVVRAHS	H257A-PVPRAHS^a^	W208Q-Q276W^a^	Mn- 6.7 keV^a^	Mn–Ca- 6.7 keV^a^	Zn–Ca^a^	Cd–Ca^a^
**A. Data collection**								
Beamline	ESRF ID29	ESRF ID29	ESRF ID29	ESRF ID23-2	ESRF ID23-1	ESRF ID23-1	ESRF ID29	ESRF ID29
Wavelength (Å)	0.97908	0.97239	0.97239	0.8726	1.85051	1.85051	1.28242	2.0664
Space group	*P6*_*3*_*22*	*P6*_*3*_*22*	*P6*_*3*_*22*	*P*6_3_22	*P*6_3_22	*P*6_3_22	*P*6_3_22	*P*6_3_22
Cell parameters – *a*, *b*, *c* (Å)	121.9 121.9 99.9	122.2 122.2 100.0	122.2 122.2 99.9	121.6 121.6 100.0	121.0 121.0 99.5	121.6 121.6 99.6	121.1 121.1 99.6	120.8 120.8 99.5
Resolution (Å)^b^	46.7–1.40 (1.42–1.40)	46.8–1.40 (1.42–1.40)	51.9–1.56 (1.59–1.56)	46.6–1.60 (1.63–1.60)	46.3–1.85 (1.89–1.85)	46.5–1.85 (1.89–1.85)	46.4–1.85 (1.88–1.85)	46.3–2.10 (2.15–2.10)
*R*_merge_ (all *I*^+^ and *I*^−^) (%)^b^^c^	9.7 (>100)	14.5 (>100)	16.2 (>100)	13.6 (>100)	5.5 (29.7)	7.0 (61.6)	7.8 (>100)	20.7 (>100)
*R*_merge_ (within *I*^+^/*I*^−^) (%)^b^^c^	–	–	15.8 (>100)	12.7 (>100)	4.6 (25.6)	6.1 (49.2)	6.6 (>100)	19.6 (>100)
*R*_meas_ (all *I*^+^ and *I*^−^) (%)^b^^d^	10.3 (>100)	15.2 (>100)	16.6 (>100)	14.6 (>100)	6.1 (39.6)	7.6 (76.9)	8.3 (>100)	21.9 (>100)
*R*_meas_ (within *I*^+^/*I*^−^) (%)^b^^d^	–	–	16.6 (>100)	14.5 (>100)	5.5 (36.1)	7.2 (67.7)	7.3 (>100)	21.8 (>100)
*R*_p.i.m._ (all *I*^+^ and *I*^−^) (%)^b^^e^	3.3 (>100)	3.5 (>100)	3.7 (>100)	5.1 (>100)	2.5 (26.0)	3.0 (45.1)	2.6 (84.1)	6.9 (58.8)
*R*_p.i.m._ (within *I*^+^/*I*^−^) (%)^b^^e^	–	–	5.2 (>100)	5.1 (>100)	3.0 (25.6)	3.8 (46.3)	3.1 (98.6)	9.4 (81.8)
CC_1/2_^b^^f^	99.9 (36.7)	99.9 (36.2)	99.9 (36.2)	99.8 (26.5)	99.8 (85.3)	99.8 (62.3)	99.9 (48.9)	99.5 (51.8)
Mean I/σ(I)^b^	10.7 (0.3)	11.6 (0.6)	12.3 (0.7)	14.1 (0.6)	22.8 (2.6)	19.5 (1.6)	18.2 (0.5)	7.6 (1.2)
Completeness (%)^b^	99.7 (96.7)	99.8 (97.2)	100.0 (100.0)	94.8 (65.4)	90.2 (35.9)	97.5 (72.5)	88.2 (34.7)	92.6 (82.4)
Multiplicity^b^	9.7 (9.3)	19.2 (17.8)	19.6 (20.2)	3.9 (1.6)	2.8 (1.2)	3.2 (1.5)	4.4 (2.6)	5.1 (4.8)
No. reflections (unique)	835040 (86228)	1678752 (87357)	1211738 (61848)	414173 (104686)	187740 (63674)	228402 (68797)	311317 (64833)	230906 (44615)
**B. Refinement**								
Resolution (Å)	46.7–1.40	46.8–1.40	51.9–1.56	46.6–1.60	46.3–1.85	46.5–1.85	46.4–1.85	46.3–2.10
*R*_work_/*R*_free_ (%)^g^	19.7/21.8	18.3/19.9	18.4/20.7	19.7/21.5	16.4/18.9	16.3/18.1	21.4/23.5	19.6/22.2
Macromolecules/a.s.u.	1	1	1	1	1	1	1	1
*No. atoms*								
Protein	2437	2433	2384	2390	2377	2358	2354	2306
Water	209	251	203	197	226	212	137	101
Ligands	74	77	93	123	125	136	132	108
*B-factor (Å*^*2*^*)*								
Protein	28.7	22.1	28.8	19.2	18.9	19.8	35.2	35.7
Water	35.7	31.1	34.6	28.0	21.0	27.5	38.8	35.6
Ligands	28.2	22.1	33.3	19.2	26.6	23.7	41.7	39.6
*r.m.s.d. from ideal values*								
Bond lengths (Å)	0.02	0.02	0.02	0.02	0.02	0.02	0.02	0.02
Bond angles (deg.)	1.4	1.4	2.1	1.4	1.4	1.4	1.4	1.9
*Ramachandran plot*								
Core region (%)	96.9	95.6	97.2	96.5	96.5	97.2	94.5	95.9
Outliers (%)	0.4^h^	0.4^h^	0.4^h^	0.4^h^	0.4^h^	0.4^h^	0.4^h^	0^h^
PDB ID	6TSL	6TSM	6YH0	6TSN	6TSQ	6TSR	6TSP	6TSO

a Friedel pairs were treated as different reflections.

b Values in parentheses refer to highest resolution shell.

c *R*_merge_ = Σ_**h**_Σ_*j*_ |*I*_**h***j*_ - ⟨*I*_**h**_⟩|/Σ_**h**_Σ_*j*_*I*_**h***j*_, where ⟨*I*_**h**_⟩ is the mean intensity of symmetry-related reflections *I*_**h**_.

d *R*_meas_ = Σ_**h**_ [N_**h**_/(N_**h**_-1)]^1/2^ Σ_*i*_ |*I*_**h***j*_ - ⟨*I*_**h**_⟩|/Σ_**h**_Σ_*i*_*I*_**h***j*_, where N is the redundancy of reflection **h**.

e *R*_p.i.m._ = Σ_**h**_ [1/(N_**h**_-1)]^1/2^ Σ_*j*_ |*I*_**h***j*_ - ⟨*I*_**h**_⟩|/Σ_**h**_Σ_*j*_*I*_**h***j*_.

f The high resolution cut-off was chosen despite the low CC_1/2_ ensuring the presence of a low signal-to-noise ratio by visual inspection of the electron density map.

g *R*_free_ was calculated from 5% of randomly selected reflections for each data set.

h One outlier at the border to the allowed region of the Ramachandran plot (Gln211) corresponding to 0.4%, characterized by well-defined electron density (r.s.c.c. = 0.99).

**Table 2:** Anomalous data collection and refinement statistics.

	Mn-6keV^a^	Mn-6.7 keV^a^	Mn–Ca-6keV^a^	Mn–Ca-6.7 keV^a^	Zn–Ca^a^	Cd–Ca^a^
**A. Data collection**						
Beamline	ESRF ID23-1	ESRF ID23-1	ESRF ID23-1	ESRF ID23-1	ESRF ID29	ESRF ID23-1
Wavelength (Å)	2.06641	1.850510	2.06641	1.85057	1.28242	2.06640
Space group	*P*6_3_22	*P*6_3_22	*P*6_3_22	*P*6_3_22	*P*6_3_22	*P*6_3_22
Cell parameters – *a*, *b*, *c* (Å)	121.0 121.0 99.5	121.0 121.0 99.5	121.7 121.7 99.6	121.7 121.7 99.6	121.4 121.4 99.8	121.0 121.0 99.6
Resolution (Å)^b^	46.4–2.40 (2.49–2.40)	45.0–2.40 (2.49–2.40)	46.6–2.20 (2.27–2.20)	45.0–2.20 (2.27–2.20)	46.5–2.25 (2.33–2.25)	46.4–2.85 (3.00–2.85)
*R*_merge_ (within *I*^+^/*I*^−^) (%)^b^^c^	7.3 (33.1)	3.5 (5.5)	6.5 (33.2)	4.3 (12.1)	4.2 (12.1)	10.9 (28.2)
*R*_meas_ (within *I*^+^/*I*^−^) (%)^b^^d^	8.8 (39.6)	4.1 (6.6)	7.7 (39.8)	5.1 (14.3)	4.6 (14.2)	12.1 (31.5)
*R*_p.i.m._ (within *I*^+^/*I*^−^) (%)^b^^e^	4.8 (21.4)	2.2 (3.6)	4.0 (21.6)	2.6 (7.6)	1.9 (5.6)	5.1 (13.7)
CC_1/2_ (%)^b^	99.6 (89.7)	99.8 (99.4)	99.7 (89.5)	99.8 (98.2)	99.9 (99.0)	99.8 (96.2)
CC_ano_ (%)^b^	8 (0)	40 (23)	13 (3)	25 (8)	59 (31)	30 (2)
DelAno between half-sets^b^	0.128 (0.011)	0.425 (0.296)	0.197 (0.069)	0.323 (0.180)	0.729 (0.325)	0.267 (0)
Mean I/σ(I)^b^	22.3 (5.4)	38.0 (26.1)	23.9 (5.1)	31.4 (13.6)	38.9 (15.4)	18.3 (9.4)
Completeness (%)^b^	99.5 (98.9)	98.9 (95.9)	99.6 (95.9)	99.5 (95.7)	99.1 (97.9)	98.1 (94.4)
Multiplicity^b^	3.2 (3.2)	3.3 (3.2)	3.5 (3.2)	3.5 (3.5)	5.2 (5.2)	9.7 (9.1)
No. reflections (unique)	104899 (31679)	104325 (31558)	150828 (41667)	151017 (41519)	214586 (38687)	99857 (18637)

a Friedel pairs were treated as different reflections.

b Values in parentheses refer to highest resolution shell.

c *R*_merge_ = Σ_**h**_Σ_*j*_ |*I*_**h***j*_ - ⟨*I*_**h**_⟩|/Σ_**h**_Σ_*j*_*I*_**h***j*_, where ⟨*I*_**h**_⟩ is the mean intensity of symmetry-related reflections *I*_**h**_.

d *R*_meas_ = Σ_**h**_ [N_**h**_/(N_**h**_-1)]^1/2^ Σ_*i*_ |*I*_**h***j*_ - ⟨*I*_**h**_⟩|/Σ_**h**_Σ_*i*_*I*_**h***j*_, where N is the redundancy of reflection **h**.

e *R*_p.i.m. ._ = Σ_**h**_ [1/(N_**h**_-1)]^1/2^ Σ_*j*_ |*I*_**h***j*_ - ⟨*I*_**h**_⟩|/Σ_**h**_Σ_*j*_*I*_**h***j*_.

### 2.6 Model building and refinement

Real space refinement of the model generated with PHASER was carried out using Coot (Emsley et al., 2010) by first removing ill-defined side chains and loop Ile53-Asn55, and subsequently adding missing structural elements in a step-wise fashion as the quality of the electron density map improved. Model building was alternated with refinement cycles using REFMAC5, refining against anomalous data when possible (Murshudov et al., 2011). The model was built by first adding the metal ions, then the sugar ligands, the water molecules and small ligands present either in the mother liquor (*e*.*g*., DMSO, sodium ions) or the cryoprotecting solution (ethylene glycol). The peptide fragments and other special features (*e*.*g*., arsenocysteine) were modeled at the end of the refinement, when the difference electron density at the active site allowed unambiguous tracing of the structural features. A list of all components included in the final models is given in Table S2. Additional reflections beyond an *R*_merge_ of 0.55 were included after carrying out the refinement of the final model using different resolution cut-offs and assessing the maps as well as CC_1/2_, as suggested by Diederichs and Karplus (Diederichs and Karplus, 2013, Karplus and Diederichs, 2015). The final model contains residues 2–293, with no electron density accountable for the first methionine residue, suggesting that it might be cleaved off during protein synthesis. The residual density at the catalytic cleft allowed modeling of a truncated peptide probe, corresponding to the first four amino acids of either the PVPRAHS or the PVVRAHS peptide. Electron density and anomalous difference maps were calculated using the program FFT, while structure factor subtraction was performed using the SFTOOL utility. Both tools are components of the CCP4 software suite for macromolecular crystallography (Winn et al., 2011). Anomalous Fourier difference maps were computed using the phases of the respective refined models. Validation of the refined models was carried out using the MolProbity server (Chen et al., 2010). Additional parameters were calculated with phenix.validate (Adams et al., 2010), and r.m.s.d. values were calculated using the PDBeFold server (Krissinel and Henrick, 2004). The refined structures were deposited in the Protein Data Bank (PDB, www.rcsb.org, (Berman et al., 2000)) with PDB IDs: 6TSL, 6TSM, 6TSN, 6TSQ, 6TSR, 6TSP and 6TSO. All figures displaying structural data were generated using PyMOL, version 1.7 (Schrödinger LLC).

## 3 Results

### 3.1 Enzyme–substrate interaction

**Fig. 2:**
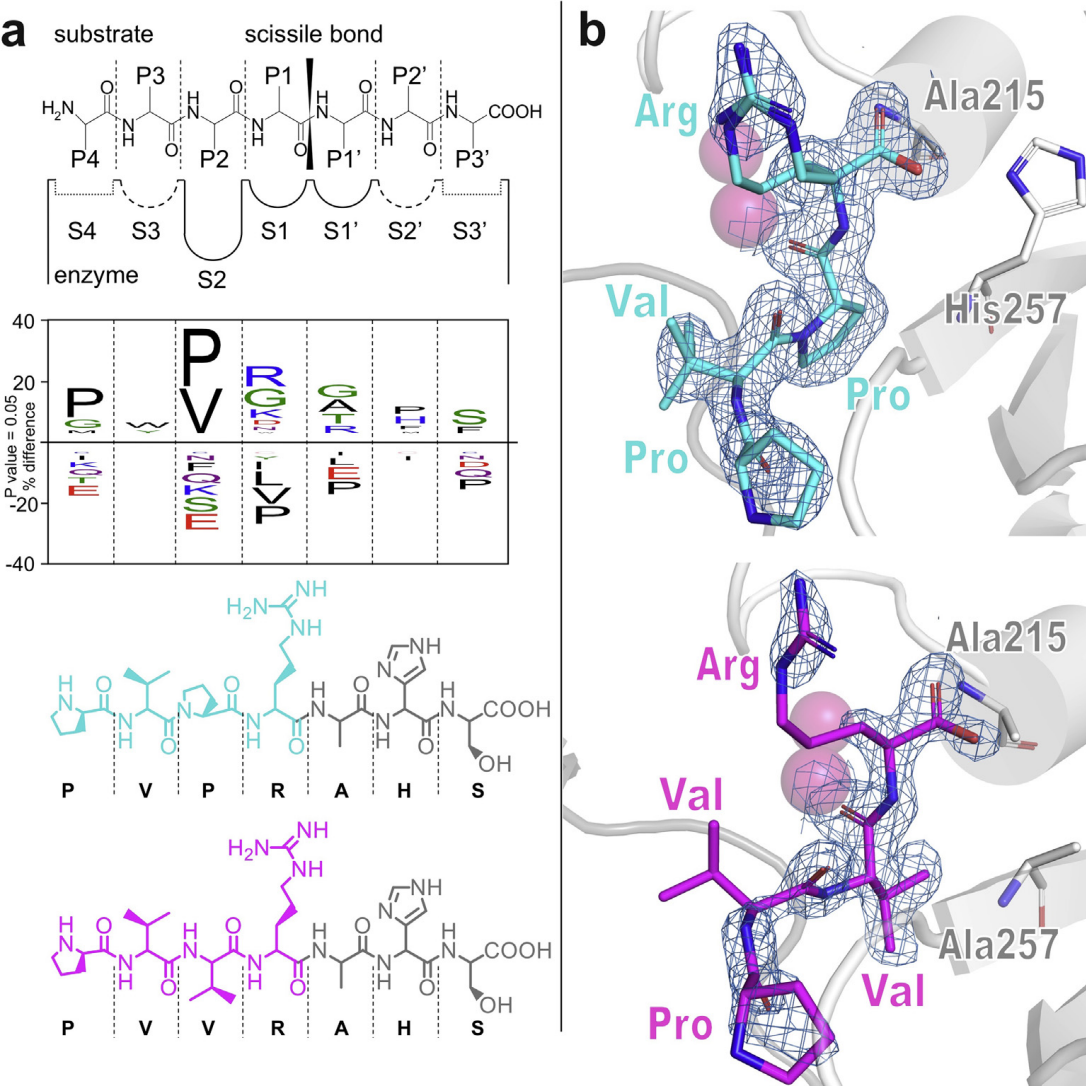
MOA complexes with substrate mimics. (a) Upper panel: Schematic representation of papain-like cysteine protease (PLCP) active site and interaction with peptide substrate, adapted from a model proposed by Brömme (2001). Middle panel: IceLogo diagram with amino acid substrate preference of MOA subsites (aligned to upper panel), as reported by Cordara et al. (2016). Bottom panels: Two synthetic peptides used for co-crystallization with MOA in this study. The peptide fragments visible in the X-ray crystal structures are highlighted in cyan/magenta. (b) Catalytic cleft of inactive MOA variants in complex with proteolytic fragments. Upper panel: MOA variant C215A co-crystallized with the peptide PVPRAHS (PDB ID: 6TSL); lower panel: MOA variant C215A/H257A co-crystallized with PVVRAHS (PDB ID: 6TSM). The electron density maps (σ_A_-weighted 2m*F*o-*F*c map contoured at 1.0 σ) only cover parts of the original peptide probes, namely PVPR (cyan) and PVVR (magenta), indicating that cleavage took place, despite using inactive MOA variants.

MOA structures in complex with irreversible inhibitors provided a first glimpse into MOA-substrate interactions (Cordara et al., 2016, Cordara et al., 2017). In an effort to validate our substrate binding model (Fig. 1c), we co-crystallized the enzymatically inactive MOA variants C215A and C215A/H257A with two synthetic heptapeptides mimicking proteolytic substrates. The designed peptides have the sequence PVxRAHS (with x = P or V). The peptide sequence was chosen based on the substrate preference (Fig. 2a) determined by Wohlschlager et al. (2011), combined with the substrate binding geometry of MOA mapped by Cordara et al. (2016) for subsites S4–S1 and S1′-S3′. The P2 (‘x’) position is occupied by either a proline or a valine residue to address the MOA S2 specificity pocket preference for both amino acids.

**Fig. 3:**
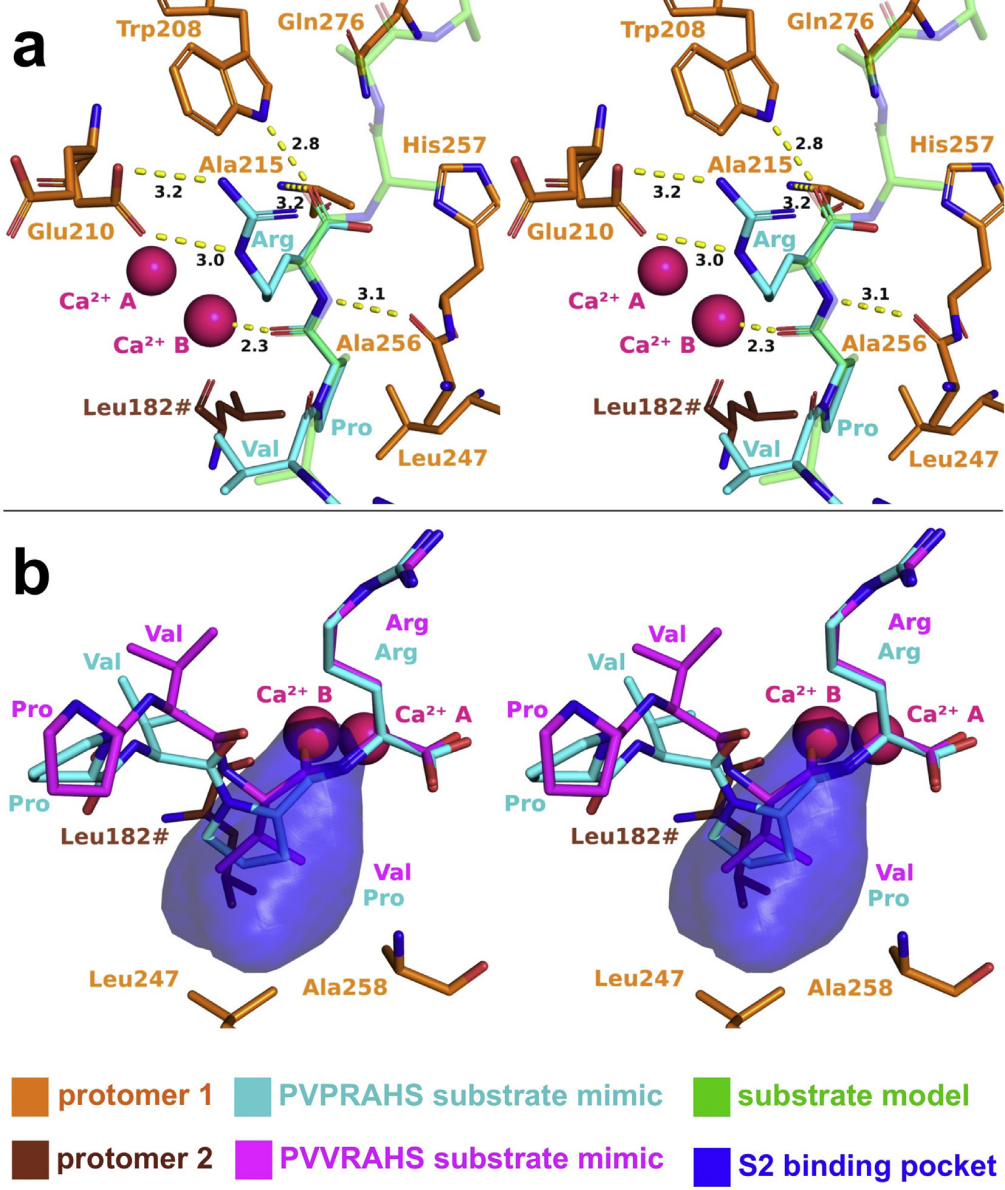
Enzyme-peptide contacts. (a) Comparison of a proposed MOA substrate binding model (light green) with the peptide structure experimentally identified in this work (PVPR, cyan), bound to MOA variant C215A (orange) (PDB ID: 6TSL). The experimental data confirm the reliability of the model derived from the structure of MOA in complex with a peptide-like inhibitor (Cordara et al., 2016), with the oxyanion hole defined by the backbone NH group of Cys215 and the site B metal ion. (b) Interaction of the P2 moiety of proteolytic fragments PVPR (cyan; PDB ID: 6TSL) and PVVR (magenta; PDB ID: 6TSM) with the S2 binding pocket of MOA (orange). The S2 binding pocket of MOA (transparent surface representation) accommodates the P2 residue of the peptides (Pro or Val, respectively) in similar conformations. Both panels show stereo-representations of the structures.

The structures of the MOA C215A-PVPRAHS and C215A/H257A-PVVRAHS complexes (PDB ID: 6TSL and 6TSM, respectively) were solved to 1.4 Å resolution. Data collection and refinement statistics are summarized in Table 1. The structures closely resemble the wild-type enzyme (r.m.s.d. = 0.12 Å for both MOA-C215A and MOA-C215A/H257A compared to PDB ID: 3EF2 (Grahn et al., 2009)), including Ramachandran plot outlier Gln211 (99.3% real-space correlation coefficient), already reported by Grahn et al. (2007). To our surprise, the co-crystallized peptide probes were cleaved in both structures, despite the fact that we used catalytically inactive enzymes (Fig. 2b). The observed cleavage products correspond to the N-terminal proteolytic fragments, binding to the MOA active site in the standard backbone orientation expected for PLCP substrates. Throughout subsites S1–S4, the peptides match the proposed MOA substrate model (Fig. 3a) derived from the MOA-bound Z-VAD-fmk inhibitor complex (Cordara et al., 2016). The synthetic peptides are anchored to the active site through three main contact points: the oxyanion hole, defined by Trp208 and the backbone NH group of Ala215, the ‘site B’ Ca^2+^ ion and a backbone–backbone interaction with Ala256 (Fig. 3). The presence of an arginine residue at the P1 position unambiguously maps the S1 subsite to the carboxylate group of L-domain Glu210, which forms a side-on bidentate salt bridge with the Arg guanidinium group (Fig. 3a). The P2 moiety binds to the S2 specificity pocket lined by residues Leu247, Ala258 and Leu182# (with ‘#’ identifying a residue provided by the other subunit; Fig. 3b). Substitution of Pro for Val at the P2 position yields a minor conformational change at the P3 and P4 positions of the peptides, and a lower occupancy of the bound proteolytic fragment (Fig. 3b).

In parallel to our own work (presented in the PhD thesis of Dipankar Manna (2016)), Sosnoswki & Turk reported a similar discovery of a trapped propeptide in catalytically inactive cathepsin L (Sosnowski and Turk, 2016). These are rare examples of proteolytic fragments observed in PLCPs.

### 3.2 Functional significance of the Trp–Gln structural swap

**Fig. 4:**
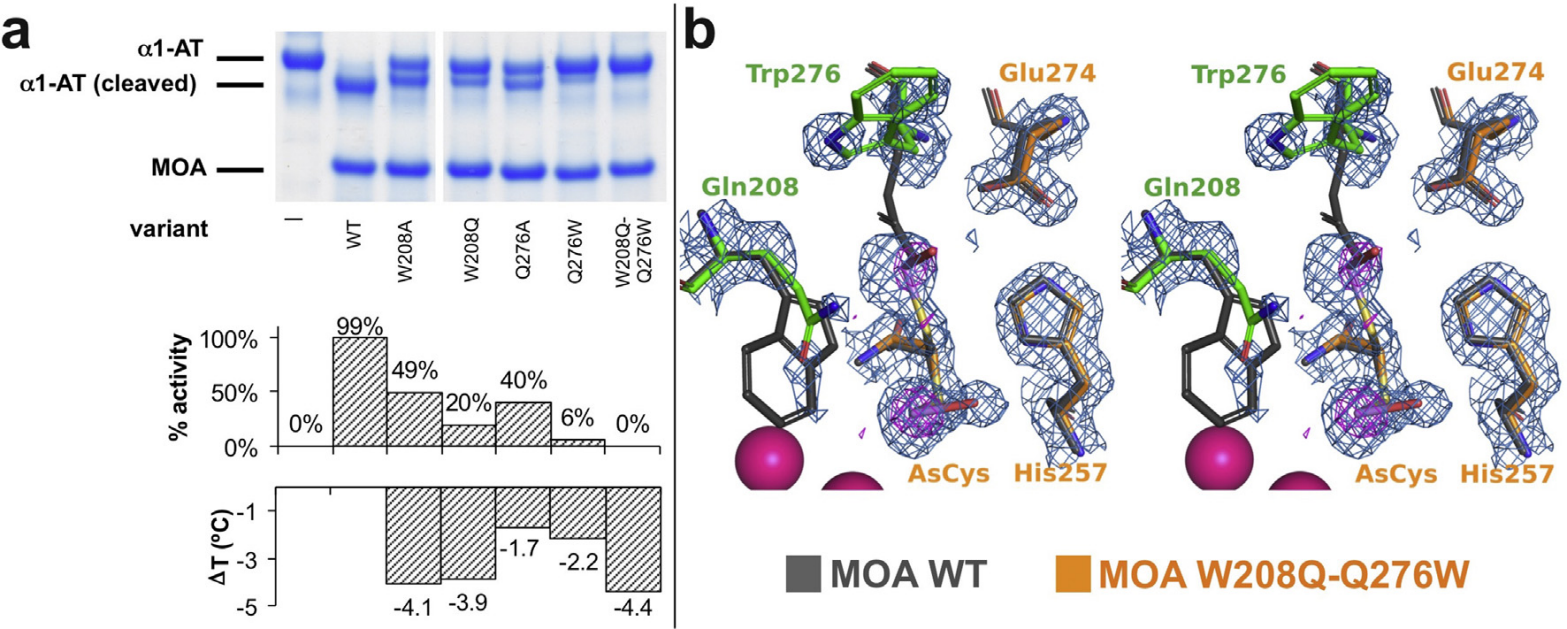
Characterization of the MOA-papain Trp/Gln structure-functional swap. (a) SDS-PAGE gel showing the digestion of a model substrate (α1-antitrypsin) with different MOA variants targeting positions 208 and 276 (lanes 3 to 7), which are involved in a structure-functional swap with Gln19 and Trp177 of papain. None of the tested variants exhibited full activity, with Q276W and W208Q/Q276W being almost completely inactive. The lower panel shows a semi-quantitative analysis of the enzyme activity as probed by Differential Scanning Fluorimetry (DSF/Thermofluor) combined with a temperature stability assay identifying Trp208 as important contributor to stability. (b) Stereo-representation of the catalytic center of MOA variant W208Q/Q276W (PDB ID: 6TSN). Consistent with the loss of activity, the σ_A_-weighted 2m*F*o-*F*c map contoured at 1.0 σ (blue mesh) shows poorly defined electron-density for Gln208 and Trp276 (green sticks). The anomalous difference density map contoured at 3.5 σ (magenta) shows peaks corresponding to the arsenic atom in the arsenocysteine species. The structure of wild-type MOA (dark grey, PDB ID: 3EF2 (Grahn et al., 2009)) is superimposed for comparison.

Compared to papain, MOA exhibits a positional and functional swap of a glutamine and a tryptophan residue (Gln19/Trp177 in papain, and Trp208/Gln276 in MOA) (Fig. 1c). To explore the significance of the structure-functional swap, we generated and tested the activity and thermal stability of both single- and double-site MOA variants targeting positions 208 and 276. The single-site substitution of Trp208 or Gln276 to either Ala (W208A, Q276A) or its papain counterpart (W208Q, Q276W) led to a partial loss of the enzymatic activity and decreased thermal stability (Fig. 4a). A MOA variant where Trp208 and Gln276 were both changed to their PLCP substitutes (W208Q/Q276W) completely lacked proteolytic activity and also had lower thermal stability than the wild-type enzyme (Fig. 4a).

The structure of the MOA W208Q/Q276W variant (PDB ID: 6TSN) was solved to 1.6 Å resolution (Table 1). While the structure is essentially identical to the wild-type enzyme (r.m.s.d. = 0.20 Å compared to PDB ID: 3EF2, chain D (Grahn et al., 2009)), the active site shows poorly defined electron density for Gln208 and Trp276 at their new positions (Fig. 4b). The Gln208 side chain amide occupies a similar position as the indole NH of Trp208 in wild-type MOA, whereas the Trp276 side chain points away from the catalytic center rather than mimicking the orientation of Trp177 in papain (compare Fig. 4b with Fig. 1c). The side chains of both residues are relatively poorly defined by electron density and exhibit higher B-factors than in the MOA wild-type structure. The catalytic cysteine (Cys215) can be modeled in two different conformations, oriented approximately 90° apart (Fig. 4b). In the crystal structure of the double mutant, the Cys215 thiol group was found to be chemically modified to arsenocysteine. The formation of an arsenocysteine has previously been reported for other thiol-based enzymes (DeMel et al., 2004, Winger et al., 2008), and is a consequence of the reaction of cysteine with cacodylate, which was used as buffering agent in the crystallization conditions.

These data show clearly that Trp and Gln are not interchangeable and fulfill different roles in papain and MOA.

### 3.3 Metal binding site occupancy: calcium versus manganese (II)

Manganese (II) was previously shown to act as functional substitute for calcium in MOA (Cordara et al., 2011). The binuclear metal cluster offers both pentahedral (site A) and octahedral (site B) coordination (Fig. 1b). In order to probe the metal binding preference for each site, we collected anomalous X-ray diffraction data on crystals grown in the presence of either manganese (II) alone or a combination of calcium and manganese (II). By choosing wavelengths on both sides of the manganese Κ absorption edge (at 6.0 keV, where only calcium shows an anomalous signal, and at 6.7 keV), we could discriminate between the presence of calcium, manganese or a mixture of the two ions. This can be accomplished by subtracting the 6.0 keV and the 6.7 keV anomalous difference maps.

**Fig. 5:**
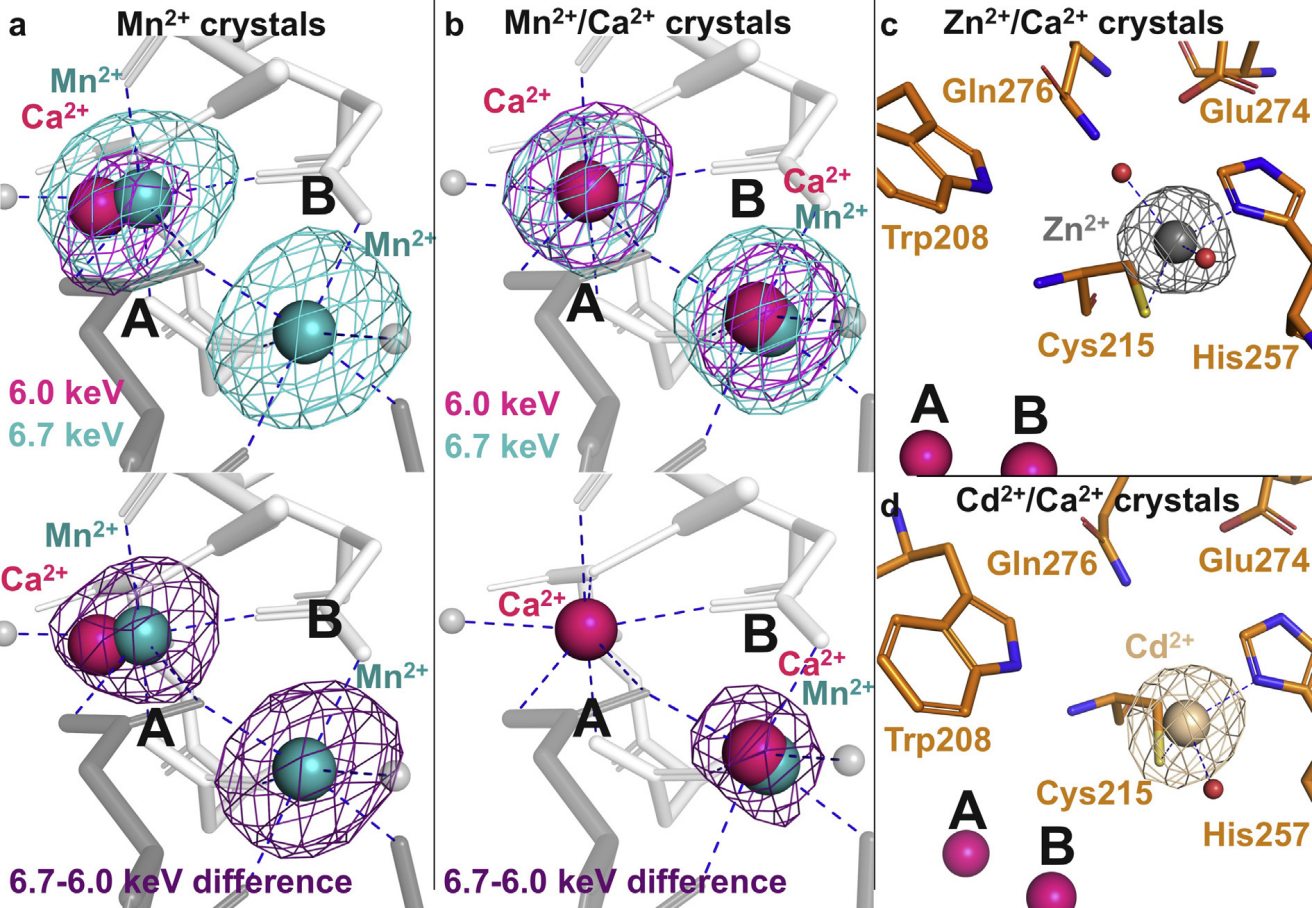
Interaction of MOA with metal ions. (a) MOA active site (amino acid residues in grey, calcium in magenta, and manganese II in cyan) with anomalous difference density maps contoured at 3.5 σ (PDB ID: 6TSQ). Note that calcium was not added to the crystallization conditions. Upper panel: maps were derived from diffraction data collected before (6.0 keV, magenta) and after (6.7 keV, cyan) the K absorption edge of manganese. The 6.0 keV map shows a single peak at site A, compatible with the presence of calcium, while the 6.7 keV map shows peaks at both sites, compatible with the presence of either ion. Lower panel: difference map generated by subtracting the two maps confirms the mixed Ca^2+^/Mn^2+^ occupancy at site A, and the exclusive presence of Mn^2+^ at site B. (b) Similar experiment compared to (a), however, with MOA crystals grown in the presence of calcium and manganese (II) (1:1 molar ratio) (PDB ID: 6TSR). Lower panel: difference map indicates exclusive presence of Ca^2+^ at site A, and a mixture of Ca^2+^ and Mn^2+^ at site B. (c and d) Structural basis of the zinc- and cadmium-based inhibition of the proteolytic activity of MOA (PDB ID: 6TSP and 6TSO, respectively). Anomalous difference density maps calculated from diffraction data collected on MOA crystals grown in the presence of zinc (c) or cadmium (d) and contoured at 4.0 σ show a large peak between the Cys–His catalytic dyad, corresponding to Zn and Cd, respectively, explaining their inhibitory effects (Maret, 2013, Shukor et al., 2006). Small grey (a,b) or red (c) spheres represent water molecules.

Phased anomalous difference maps revealed that manganese can occupy one or both of the metal binding sites, depending on the presence and relative concentration of calcium (Fig. 5a and b). As expected (McPhalen et al., 1991, Strynadka and James, 1991), site A has a strong preference for calcium, which partially occupies the pentahedral cavity even when present as a trace contaminant (approximately 0.33/0.67 calcium/manganese relative occupancy in manganese-only crystals, PDB ID: 6TSQ; Fig. 5a) and dominates completely at higher concentration (manganese/calcium-crystals, PDB ID: 6TSR; Fig. 5b). Site B is less selective. When present at equimolar concentration, the octahedral cavity is occupied by both ions, at approximately 0.75/0.25 relative occupancy (calcium/manganese; Fig. 5b).

### 3.4 Zinc and cadmium inhibition

Zinc and cadmium strongly inhibit the proteolytic activity of MOA and papain (Cordara et al., 2011, Shukor et al., 2006). It has been suggested that zinc binds at the catalytic center of PLCPs, directly coordinating the cysteine and histidine side chains of the catalytic dyad (Maret, 2013). To provide a structural basis for the inhibition, we collected anomalous diffraction data on MOA crystals grown in the presence of either zinc or cadmium. Indeed, crystals containing zinc in their mother liquor showed a large peak (>20 σ) between Cys215 and His257 in phased anomalous difference maps calculated from data collected close to the K absorption edge of zinc (9.7 keV) (Fig. 5c). The anomalous difference maps of cadmium-containing crystals (calculated from data collected at 6.0 keV) show a similar feature, consistent with the previously suggested inhibition mechanism (Maret, 2013).

## 4 Discussion

**Fig. 6:**
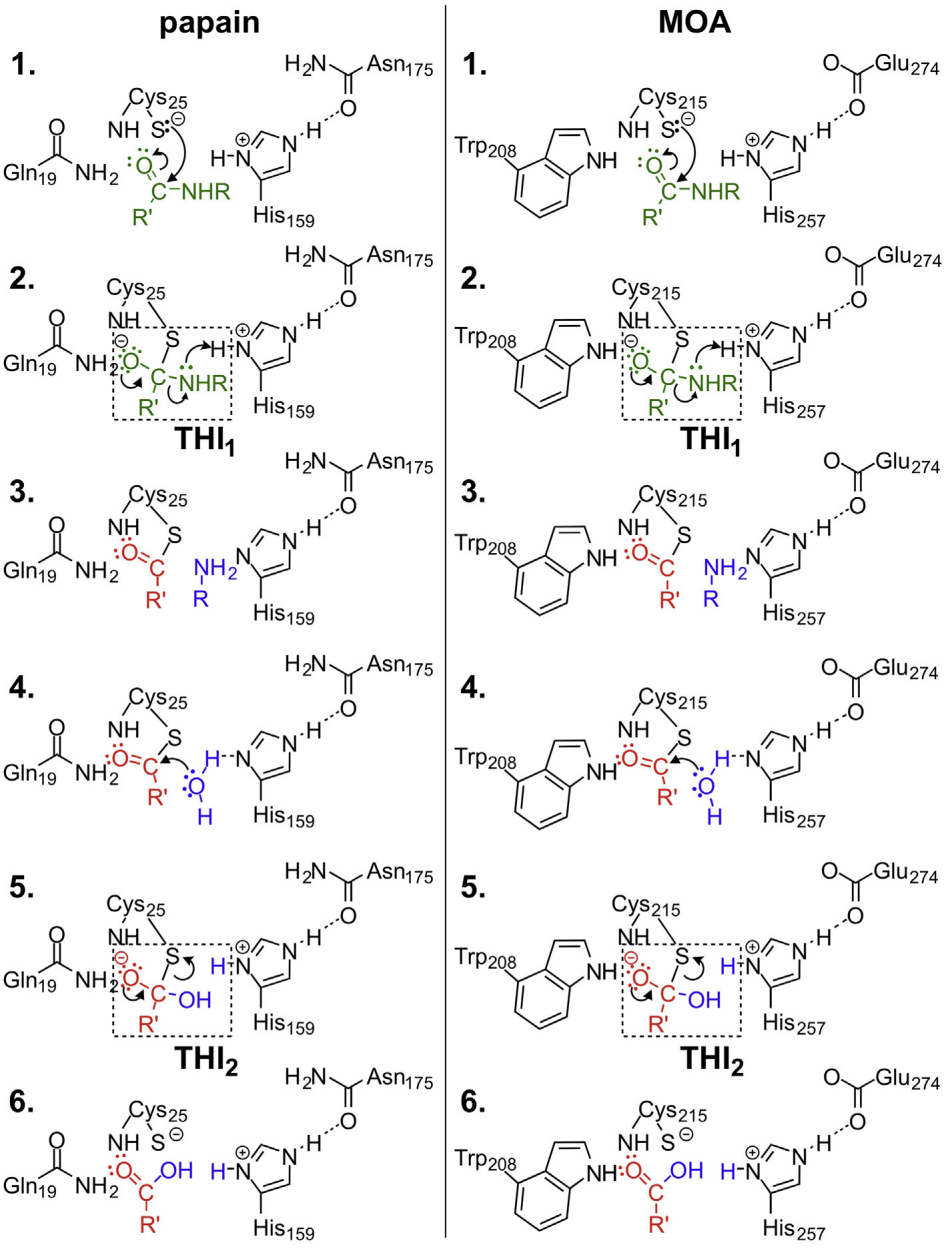
Proteolytic mechanisms of papain and MOA. Left panel: reaction mechanism derived for papain, as described by Storer and Ménard (Storer and Ménard, 1994). Acylation: the thiol group of the catalytic cysteine performs a nucleophilic attack on the carbonyl carbon (1, substrate in green), leading to cleavage of the scissile bond and formation of a tetrahedral intermediate (THI1), stabilized by the oxyanion hole (2). After proton transfer from the catalytic histidine, a thioester is formed (red) and the C-terminal fragment (blue) is released from the active site as the first reaction product (3). Deacylation: a water molecule (blue) activated by the catalytic histidine performs a nucleophilic attack on the thioester (4), leading to the formation of a second tetrahedral intermediate (THI2; 5). The reaction is completed after breakage of the thioester bond and the formation of a new carboxy-terminus on the N-terminal portion of the cleaved substrate (6, fragment in red), preparing the enzyme for a new catalytic cycle. Right panel: the same mechanism as depicted on the left, employing components from the catalytic machinery of MOA. Notably, Trp208 replaces Gln19 as an oxyanion hole component, and the catalytic histidine switches from being anchored to an asparagine to a glutamate residue (Glu274).

Cysteine proteases of the papain superfamily hydrolyze peptides by a two-step acylation-deacylation mechanism (Storer and Ménard, 1994). A Cys–His dyad lies at the heart of their catalytic machinery. Acylation starts with the nucleophilic attack by the catalytic cysteine on the substrate and ends with formation of a cysteine-substrate thioester. In the second step, the C-terminal portion of the cleaved substrate is released from the active site (Fig. 6). During the deacylation reaction, a histidine-activated water molecule hydrolyzes the thioester, leading to the formation of the new carboxy-terminus and the release of the N-terminal portion of the substrate. Both the acylation and deacylation reactions pass through a negatively charged tetrahedral intermediate (THI) stabilized by the oxyanion hole, an active site cavity commonly lined by polar NH groups (Fig. 6).

### 4.1 Preservation of the papain catalytic machinery in MOA

The Cys–His catalytic dyad is conserved in MOA, represented by residues Cys215 and His257. The reactivity of Cys215 has been confirmed by the lack of activity observed in MOA variants C215A and C215S (Cordara et al., 2011, Wohlschlager et al., 2011). Additionally, Cys215 can be found oxidized or covalently bound to proteolytic activity inhibitors or buffer components (*e.g.*, cacodylate) in various X-ray crystal structures (e.*g*., PDB IDs: 2IHO, 3EF2, 5D61, 5MU9 (Grahn et al., 2007, Grahn et al., 2009, Cordara et al., 2017, Cordara et al., 2016)), including those presented here. Finally, we showed in this work that complex formation of the catalytic dyad with metal ions like zinc or cadmium inactivates the enzyme. In papain, histidine is positioned for catalysis by Asn175, a residue often mentioned as part of a ‘*catalytic triad*’ together with Cys25 and His159. Asn175 is important, albeit non-essential in the catalytic process (Vernet et al., 1995). The substitution of asparagine with a different residue (*e*.*g*., Glu274 in MOA) is not uncommon among PLCPs (*e.g.*, streptopain B from *Streptococcus pyogenes*). While the general architecture, catalytic residues and inhibition mechanism of PLCPs appear to be conserved in MOA, its proteolytic domain carries unique features, setting it apart from other papain-like proteases.

### 4.2 The active site of MOA exhibits special features

In addition to the catalytic dyad or triad, papain and other proteases stabilize the tetrahedral intermediate through a structural feature called the ‘oxyanion hole’, which often consists of an active site cavity lined by several NH groups. In papain, the side chain of Gln19 and the backbone NH group of the catalytic cysteine assume this role (Fig. 1c); in MOA, the function of Gln19 is adopted by Trp208. Interestingly, a neighboring glutamine (Gln276) is in a structurally similar position as papain residue Trp177, assisting substrate coordination and shielding part of the active site from solvent (Storer and Ménard, 1994, Gul et al., 2008). Substitution of either residue with alanine led to a decrease in enzymatic activity and concomitant decrease in stability (Fig. 4a). The functional data presented here validate Trp208 and Gln276 in the roles assigned by their papain homology, representing a functional Trp–Gln swap. However, simply substituting both Trp208 and Gln276 for their papain counterparts in variant W208Q/Q276W was insufficient to preserve enzyme activity (Fig. 4). Taken together with the low amino acid (<18 %) and gene (<4 %) sequence identity compared to other PLCPs, this suggests more distant evolutionary origins. Indeed, a phylogeny analysis of papain-like proteases places MOA on a separate clade of the phylogenetic tree, well distinct from LapG and calpain, the only other two calcium-binding PCLPs (Supplementary Materials; Fig. S2). This notion is reinforced by the involvement of metal ions in catalysis, as further discussed below.

### 4.3 Role of metal ions in MOA

Metal binding to the MOA active site triggers a large conformational change, prealigning the catalytic machinery, vacating the oxyanion hole and removing a gating tyrosine residue from the catalytic center (Cordara et al., 2017). This mechanism has been suggested to function as an alternative to the propeptide-processing activation mechanisms of other PLCPs (Cordara et al., 2017, Khan and James, 1998). Calcium-based activation is not unique to MOA and its orthologs, and has also been observed in other proteases assigned to the papain family (*e*.*g*., calpain or LapG (Campbell and Davies, 2012, Chatterjee et al., 2012)). Moreover, the single calcium binding site in LapG superimposes well with site A in MOA (calcium binding in calpains differs). However, to our knowledge no other PLCP shows direct interaction of a metal ion with the substrate, as observed for MOA's metal site B. Our anomalous diffraction data demonstrate that site B, which adopts an octahedral coordination, is promiscuous and can feature metal ions other than calcium, *e*.*g*., manganese (II). This promiscuity, however, comes with a price in terms of catalytic activity (Cordara et al., 2011). Decreased activity may result from a difference in the ionic radii for calcium and manganese (II), yielding a less favorable interaction geometry and faster exchange with the solvent. Alternatively, as postulated for other enzymes (Snijder et al., 2001), the ions may affect the stability of the oxyanion hole, which is 5 Å away from site B. Interestingly, residue Gln211, which directly coordinates the site A metal ion with its backbone carbonyl group (Fig. 1b), exhibits somewhat strained torsion angles in the metal complex and relaxes to a more favorable geometry in high-resolution calcium-free structures of MOA (unpublished). While previously discounted as a consequence of its surface exposure, the structural tension released upon transitioning between the metal-free and metal-bound forms suggests that Gln211 might act as a ‘molecular spring’, facilitating the switch between active and inactive states upon metal ion release.

### 4.4 MOA caught in flagranti with proteolysis product

Unexpectedly, co-crystallization of catalytically inactive MOA variants with different substrate mimics resulted in complexes of the enzyme with cleaved peptides. Contamination by wild-type MOA can be excluded, since we used dedicated equipment for protein purification, and mass-spectrometric (MS) analysis of the purified proteins used for crystallization did not indicate the presence of contaminant proteases from the expression host (data not shown). The presence of smaller fragments of the substrate mimic in the original preparation was excluded by MS analysis provided by the manufacturer.

Substrate activation by the oxyanion hole combined with water-catalyzed hydrolysis is a known source of residual activity in proteases (Carter and Wells, 1988, Corey and Craik, 1992, Krishnan et al., 2000) and has also been observed in PLCPs (Sosnowski and Turk, 2016). Coupled to the long time needed to grow the crystals (2–3 weeks), this could explain the presence of a cleaved peptide rather than the full substrate. The peptide fragment occupies the S1–S4 subsites of the catalytic cleft and matches the N-terminal part of the expected proteolytic product, representing a snapshot of MOA just after the acylation step, mediated by a water molecule in the absence of the Cys nucleophile (Fig. 6 frame 3). A stretch of poorly defined electron density close to subsites S1′-S3′ suggests the lingering presence of the C-terminal fragment of the cleaved substrate. Overall, the structural and functional data fit the acylation/deacylation PLCP reaction paradigm, supporting its conservation in MOA.

In contrast to MOA variant C215A/H257A, we did not observe any traces of the peptide in wild-type MOA crystals grown under similar conditions, even at low σ levels (1.6 Å structure; not shown); and MOA variant H257A co-crystallized with PVPRAHS (1.6 Å structure with PDB ID: 6YH0; Table 1) only showed very weak and discontinuous difference density of the peptide fragment in the catalytic cleft. The lack of substrate bound to the wild-type enzyme could be a consequence of an oxidized catalytic thiol or its reaction with cacodylate, as observed in the Mn-bound structures. However, MOA was previously crystallized in complex with irreversible inhibitors at full occupancy (*e*.*g*., PDB IDs 5MU9 and 5D63 (Cordara et al., 2017, Cordara et al., 2016)), suggesting that inactivation of Cys215 is unlikely. Given that the MOA C215A variant was crystallized with a peptide bound at fully occupancy, we suggest as alternative explanation that the fully competent wild-type enzyme (but not the H257A variant), quickly and effectively expelled the substrate after processing. Complementary to its role as a nucleophile, and in line with our observation of two alternative conformations of this residue (data not shown), Cys215 may act as a molecular lever, “kicking out” the cleaved substrate.

## 5 Conclusions

MOA is a chimerolectin/protease hybrid representative of a largely unexplored family of metal-dependent fungal enzymes (Cordara et al., 2017). This enzyme family carries a unique papain-like domain, which also acts as dimerization domain. The proteolytic domain of MOA shows both shared and diverging features compared to other PLCP members. The oxyanion hole and catalytic dyad are common to other enzymes of the papain superfamily, while divergent features include a calcium/manganese (II)-dependent tyrosine-gated activation mechanism and a direct interaction between the bound metal ions and the substrate. The structural data presented in this work consolidate the catalytic role assigned to key active site determinants of MOA (oxyanion hole, catalytic dyad) in previous publications (Cordara et al., 2011, Cordara et al., 2016, Wohlschlager et al., 2011). Moreover, we caught MOA *in the act*, in complex with proteolytic fragments.

The structures of MOA in complexes with cleaved peptides presented here are consistent with the acylation/deacylation reaction mechanism accepted for proteases of clan CA, the papain-like superfamily of peptidases (Storer and Ménard, 1994). Throughout a given superfamily of enzymes, a common structural strategy is employed to stabilize similar catalytic intermediates. This involves conserved functional groups devoted to specialized tasks. The combination of shared chemistry and conserved framework serve as identifying criteria to assign new members to that family (Babbitt and Gerlt, 1997). The common structural features and conserved reaction mechanism firmly establishes MOA and its orthologs as members of clan CA. However, the low sequence conservation and characteristic structural motifs of these chimerolectins point to a separate evolutionary origin, and convergent evolution towards a fungal-specific embodiment of the papain fold. The unique role of metal ions in catalysis, tyrosine gating, and structural differences such as the Trp/Gln swap set MOA apart from other papain-like proteases, defining a new subfamily with its own molecular identity.

## Author contributions

DM and GC contributed equally to this work. GC and UK conceived the study, DM and GC performed the experiments and analyzed the data. UK supervised DM and GC, and validated the structures. DM and GC drafted the manuscript, which was revised and finalized together with UK.

## Declaration of Competing Interest

The authors declare that they have no conflict with the contents of this article.

## Abbreviation list

α1-AT: α1-antitrypsin from human plasma
MOA: *Marasmius oreades* agglutinin
MS: mass spectrometry
PLCPs: papain-like cysteine proteases
r.m.s.d.: root mean square deviation/difference
r.s.c.c.: real-space correlation coefficient
THI: tetrahedral intermediate
