# Supplementary material for: Crystal structure of MOA in complex with a peptide fragment: A protease caught in flagranti
Source: Curr Res Struct Biol. 2020 Apr 22;2:56–67. doi: 10.1016/j.crstbi.2020.04.003 (PMC8244254; doi:10.1016/j.crstbi.2020.04.003)
Supplement: Multimedia component 1 [file mmc1.pdf]

Supplementary data

Crystal structure of MOA in complex with a peptide fragment: a protease caught *in flagranti*

Dipankar Manna[1†], Gabriele Cordara[1† *], and Ute Krengel[1*]

[1]*Department of Chemistry, University of Oslo, PO Box 1033 Blindern, 0315 Oslo, Norway*

[†]*contributed equally to this work*

**corresponding authors: gabriele.cordara@kjemi.uio.no, Tel.: +47 22855464; ute.krengel@kjemi.uio.no, Tel.: +47 22855461*

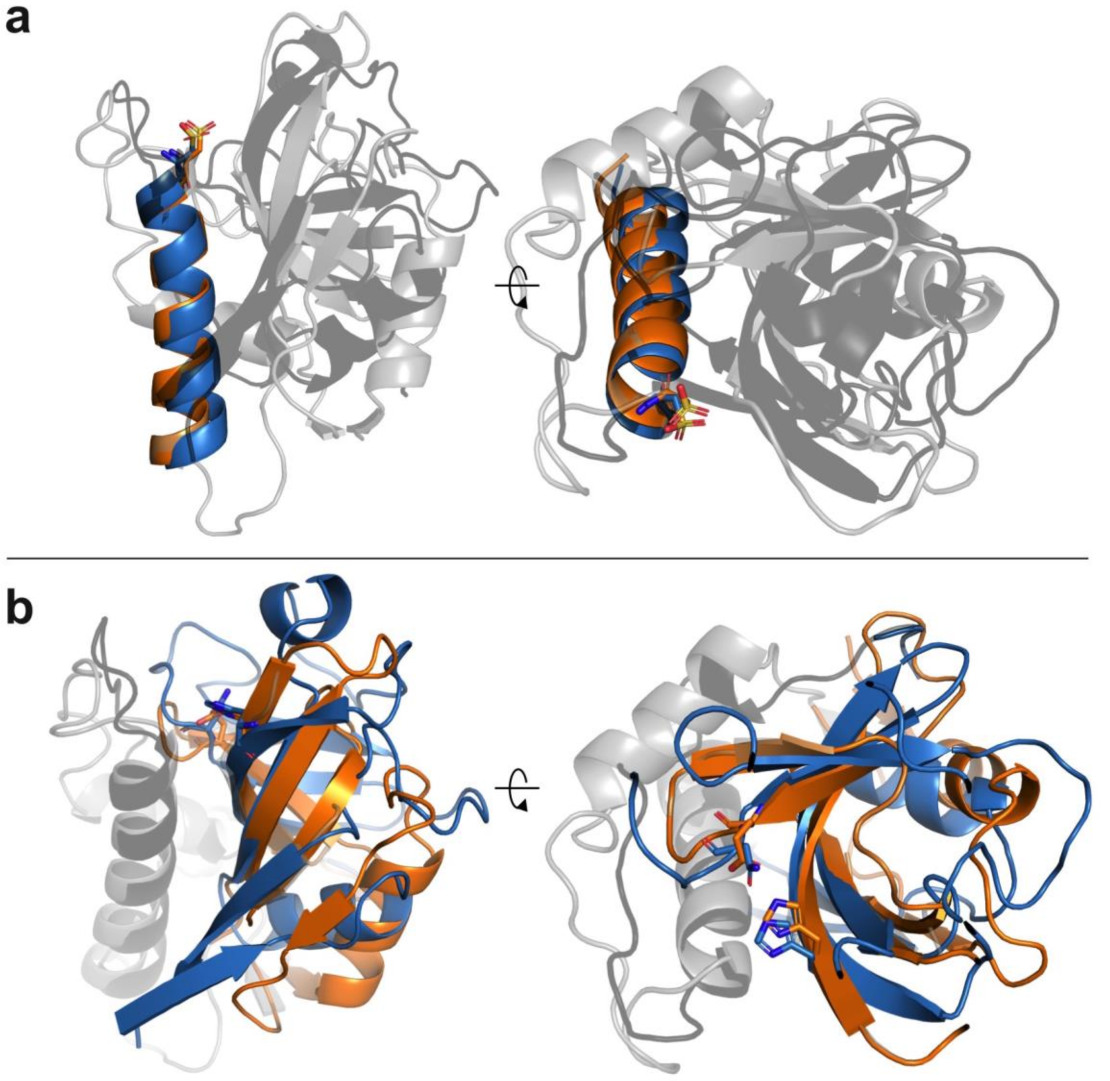

Figure S1 – Secondary Structure Matching (SSM) superposition of MOA and papain. The proteolytic domain of MOA (amino acids 156-293, PDB ID: 3EF2 [1], light grey and orange) has been matched to the structure of the papain proteolytic core (amino acids 1-43 and 108-212, PDB ID: 9PAP [2], dark grey and azure) using the SSM algorithm implemented in *Coot* [3]. The conservation of the catalytic cysteine-carrying α–helix and the catalytic histidine-carrying β–barrel is highlighted in panels **a** and **b**, respectively. In both structures the thiol group of the catalytic cysteine is oxidized to a sulfone.

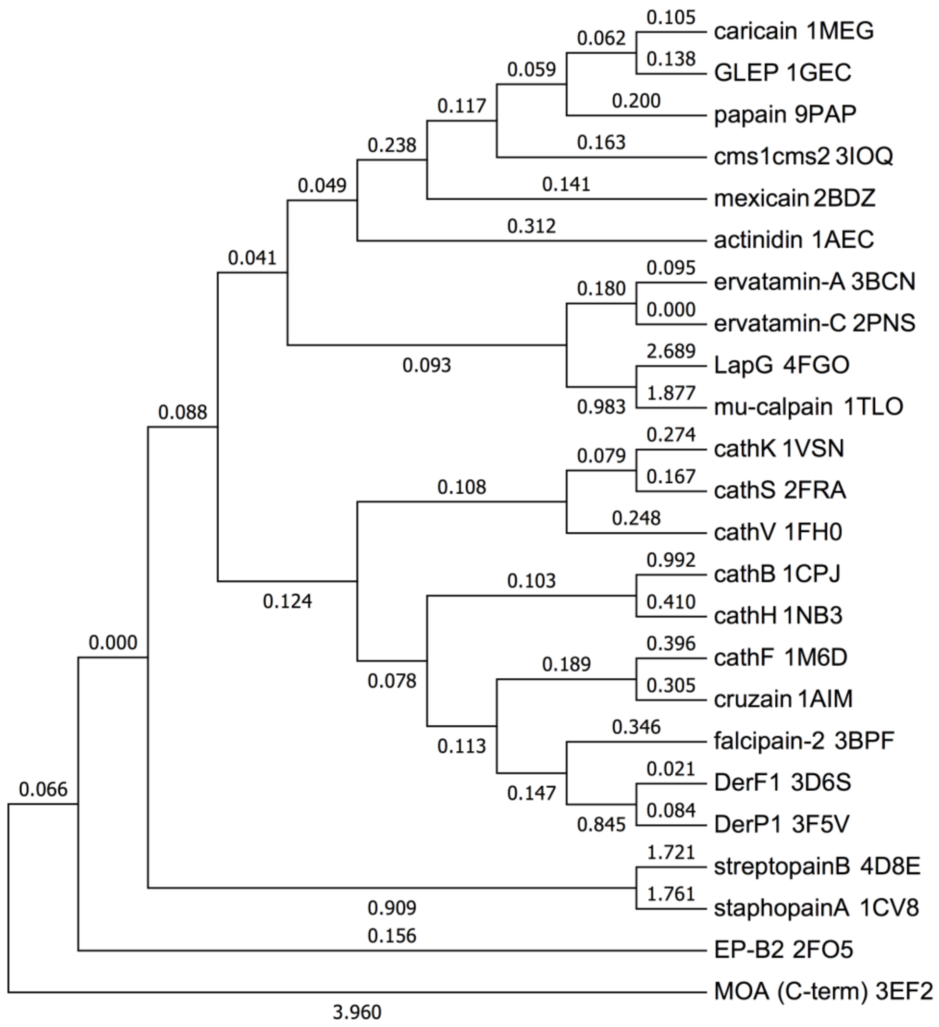

Fig. S2 Phylogenetic analysis by Maximum Likelihood method. Amino acid sequences of the proteolytic domain of MOA and 23 papain-like cysteine proteases were aligned using ClustalW [4]. Alignment targets were selected from the top-ranking PLCP hits of a DALI search [5], carried out using the coordinates of the Ca^{2+}-bound MOA (PDB ID: 3EF2 [1]). Evolutionary analyses were performed with MEGA7 [6]. The evolutionary history was inferred by the Maximum Likelihood method based on the JTT matrix-based model [7]. The tree with the highest log likelihood is shown, with each branch reporting the number of substitutions per site and the PDB ID of the aligned targets.

Table S1 – Components of the crystallization solutions

Data set	MOA variant	protein [C] (mg/ml)	blood group B type 2 trisaccharide (protein:sugar molar ratio)	peptide ligand (protein:peptide molar ratio)	Crystallization solution components
PVPRAHS	Cys215Ala	5	1:20	1:10	0.1 M Na-acetate pH 5.0, 18 % PEG 8000, 0.25 M sodium acetate, 10 mM $CaCl_2$, 5 mM DTT
PVVRAHS	Cys215Ala-His257Ala	5	1:20	1:10	0.1 M Na-cacodylate/HCl pH 6.5, 20 % PEG 8000, 0.2 M sodium acetate, 10 mM $CaCl_2$, 5 mM DTT
WT-Cys	His257Ala	5	1:20	1:10	0.1 M cacodylate pH 6.5, 0.2 M sodium acetate, 22% PEG 8000, 10 mM $CaCl_2$, 5 mM DTT
W208Q-Q276W	Trp208Gln-Gln276Trp	5	1:20	-	0.1 M cacodylate/HCl pH 6.5, 14 % PEG 8000, 0.2 M calcium acetate, 10% DMSO, 5 mM DTT
Mn-6keV and 6.7keV	wild-type	5	1:20	-	0.1 M cacodylate/HCl pH 6.5, 16 % PEG 8000, 0.15M sodium acetate, 5 mM $MnCl_2$, 12.5 % DMSO, 5 mM DTT
Mn-Ca-6keV and 6.7keV	wild-type	5	1:20	-	0.1 M cacodylate/HCl pH 6.5, 16 % PEG 8000, 0.15M sodium acetate, 10 mM $MnCl_2$, 10 mM $CaCl_2$, 12.5 % DMSO, 5 mM DTT
Zn-Ca	wild-type	5	1:20	-	0.1 M cacodylate/HCl pH 6.5, 16 % PEG 8000, 0.5 M sodium acetate, 50 mM $ZnCl_2$, 10 mM $CaCl_2$, 12.5 % DMSO, 5 mM DTT
Cd-Ca	wild-type	5	1:20	-	0.1 M cacodylate/HCl pH 6.5, 16 % PEG 8000, 0.2 M sodium acetate, 1 mM $CdCl_2$, 10 mM $CaCl_2$, 12.5 % DMSO, 5 mM DTT

Table S2 – Modelled features

Data set	MOA variant	sugar binding sites			metal binding cluster		catalytic cleft	Cys215 modifications
		α (res. 20-48)	β (res. 72-100)	γ (res. 123-151)	site A	site B		
PVPRAHS	Cys215Ala	y	-	y	Ca^{2+}	Ca^{2+}	PVPR peptide	- (Cys215Ala variant)
PVVRAHS	Cys215Ala-His257Ala	y	-	y	Ca^{2+}	Ca^{2+}	PVVR peptide	- (Cys215Ala variant)
WT-Cys	His257Ala	y	y (Gal-α1,3Gal)	y	Ca^{2+}	Ca^{2+}	PVPR peptide	arsenocysteine
W208Q-Q276W	Trp208Gln-Gln276Trp	y	y	y	Ca^{2+}	Ca^{2+}	-	arsenocysteine
Mn-6keV and 6.7keV	wild-type	y	y	y	Ca^{2+}/Mn^{2+}	Mn^{2+}	ethylene glycol bound to site B	arsenocysteine
Mn-Ca-6keV and 6.7keV	wild-type	y	y	y	Ca^{2+}	Ca^{2+}/Mn^{2+}	ethylene glycol bound to site B	arsenocysteine
Zn-Ca	wild-type	y	y	y	Ca^{2+}	Ca^{2+}	Zn^{2+} ion bound to the catalytic dyad<br>ethylene glycol bound to site B	-
Cd-Ca	wild-type	y	y	y	Ca^{2+}	Ca^{2+}	Cd^{2+} ion bound to the catalytic dyad<br>ethylene glycol bound to site B	-

1. Grahn EM, Winter HC, Tateno H, Goldstein IJ, Krengel U: **Structural characterization of a lectin from the mushroom *Marasmius oreades* in complex with the blood group B trisaccharide and Calcium**. *J. Mol. Biol.* 2009, **390**:457-466.
2. Kamphuis IG, Drenth J, Baker EN: **Thiol proteases. Comparative studies based on the high-resolution structures of papain and actinidin, and on amino acid sequence information for cathepsins B and H, and stem bromelain**. *J. Mol. Biol.* 1985, **182**:317-329.
3. Emsley P, Lohkamp B, Scott WG, Cowtan K: **Features and development of *Coot***. *Acta Crystallogr. D Biol. Crystallogr.* 2010, **66**:486-501.
4. Larkin MA, Blackshields G, Brown NP, Chenna R, McGettigan PA, McWilliam H, Valentin F, Wallace IM, Wilm A, Lopez R, et al.: **Clustal W and Clustal X version 2.0**. *Bioinformatics* 2007, **23**:2947-2948.
5. Holm L: **Benchmarking fold detection by DaliLite v.5**. *Bioinformatics* 2019, **35**:5326-5327.
6. Kumar S, Stecher G, Tamura K: **MEGA7: Molecular Evolutionary Genetics Analysis version 7.0 for bigger datasets**. *Mol. Biol. Evol.* 2016, **33**:1870-1874.
7. Jones DT, Taylor WR, Thornton JM: **The rapid generation of mutation data matrices from protein sequences**. *Comput. Appl. Biosci.* 1992, **8**:275-282.
